# Multiparameter Telemetry as a Sensitive Screening Method to Detect Vaccine Reactogenicity in Mice

**DOI:** 10.1371/journal.pone.0029726

**Published:** 2012-01-19

**Authors:** Margarete Arras, Daniel L. Glauser, Paulin Jirkof, Andreas Rettich, Benjamin Schade, Paolo Cinelli, Daniel D. Pinschewer, Mathias Ackermann

**Affiliations:** 1 Institute of Laboratory Animal Science, University of Zurich, Zurich, Switzerland; 2 Division of Surgical Research, Department of Surgery, University Hospital Zurich, Zurich, Switzerland; 3 Institute of Virology, University of Zurich, Zurich, Switzerland; 4 Institute of Veterinary Pathology, University of Zurich, Zurich, Switzerland; 5 Department of Pathology and Immunology, World Heath Organization Collaborating Centre for Neonatal Vaccinology, University of Geneva, Geneva, Switzerland; Instituto Butantan, Brazil

## Abstract

Refined vaccines and adjuvants are urgently needed to advance immunization against global infectious challenges such as HIV, hepatitis C, tuberculosis and malaria. Large-scale screening efforts are ongoing to identify adjuvants with improved efficacy profiles. Reactogenicity often represents a major hurdle to the clinical use of new substances. Yet, irrespective of its importance, this parameter has remained difficult to screen for, owing to a lack of sensitive small animal models with a capacity for high throughput testing. Here we report that continuous telemetric measurements of heart rate, heart rate variability, body core temperature and locomotor activity in laboratory mice readily unmasked systemic side-effects of vaccination, which went undetected by conventional observational assessment and clinical scoring. Even minor aberrations in homeostasis were readily detected, ranging from sympathetic activation over transient pyrogenic effects to reduced physical activity and apathy. Results in real-time combined with the potential of scalability and partial automation in the industrial context suggest multiparameter telemetry in laboratory mice as a first-line screen for vaccine reactogenicity. This may accelerate vaccine discovery in general and may further the success of vaccines in combating infectious disease and cancer.

## Introduction

Vaccination represents one of the most successful strategies to control or even eradicate infectious diseases. New approaches to vaccination are, however, needed to better approach global challenges such as malaria, tuberculosis, human immunodeficiency virus (HIV) and cancer. Unlike live and vectorized vaccines, inactivated ones based on whole pathogens or antigenic subunits thereof generally require the addition of adjuvants for inducing protective immune response. Aluminium hydroxide and aluminium phosphate adjuvants (alum) have been used for decades in many such vaccines, despite occasional toxic side-effects [1]–[4] and – depending on the context and the immune effector pathways analyzed – only limited efficacy. Apart from protein-based vaccines also DNA-based strategies [5], [6] require refined adjuvant systems for optimal efficacy [7]–[12]. In recent years, much effort has therefore been devoted to the search for new adjuvants with superior efficacy and safety [8], [13], [14], and some of them have successfully made it to the clinic. Yet, the need for new adjuvants and vaccine formulations in general remains a priority in combating diseases where existing strategies have failed to provide protection [10], [15]. In the eyes of many scientists, a systematic global large-scale approach provides the highest likelihood of success in this direction [16]. Such an endeavour will, however, require the development of refined and standardized screening procedures, both for vaccine efficacy as well as for safety and reactogenicity.

Current standards in safety testing include clinical scoring for symptoms of disease, investigation of body fluids (e.g. blood, urine, faeces) and post mortem pathological examination of several organs. Thus, teratogenicity, carcinogenicity and organ-specific long-term toxicity of test compounds are best detected. Irrespective of the considerable number of experimental animals required and the financial resources dedicated to the task [17], [18], the information obtained remains incomplete. Less severe reactogenicity can go undetected but may still render many compounds inappropriate for clinical use. Thus, the need for novel approaches in preclinical safety screening of adjuvants has become increasingly recognized in recent years [15], and includes notably a requirement for objective measures of short-term reactogenicity, which is not commonly born out in long-term health impairment i.e. teratogenicity, carcinogenicity or persisting histopathological alterations.

Here we have exploited multiparameter telemetry as a novel preclinical method for the assessment of vaccine reactogenicity. Our approach allows the evaluation of test compounds using a limited number of laboratory mice. We show that the continuous and combined telemetric measurements of heart rate, heart rate variability, body core temperature and locomotor activity is well suited to detect transient reactogenicity of adjuvants ranging from sympathetic activation over pyrogenicity to apathy. These parameters therefore not only detect substantial health impairment but also record signs of minor distress, both in an investigator-independent and accurate way. Standardized results in real-time allow for rapid decision-making in the context of screening projects. This technique will therefore facilitate the rapid and accurate selection of promising candidate adjuvants and vaccine formulations, and the early exclusion of reactogenic compounds. Multiparameter telemetry can thus help saving financial resources and will speed up the selection and development of vaccination approaches with promising safety and tolerability profiles.

## Results

### Experimental design

**Figure 1:**
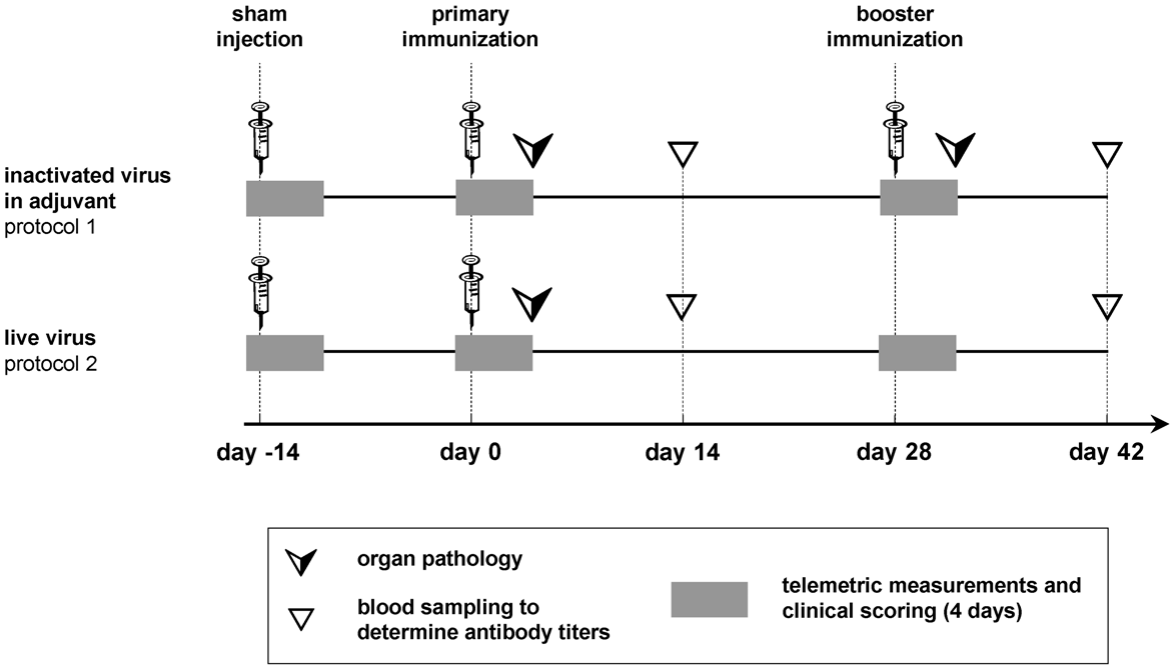
Experimental design. Eight mice per immunization protocol were equipped with telemetric transmitters for real-time, long-term recording of heart rate, interbeat interval, heart rate variability (standard deviation of interbeat interval), body core temperature and locomotor activity. Simultaneous clinical scoring included body weight measurements, as well as food and water consumption. Measurements were conducted over a period of four days (grey), the first of which served as individual baseline. Antibody responses were determined in serum samples collected two weeks after primary and booster immunization, respectively (triangles). Twenty-two mice without transmitter were treated analogously and were sacrificed for gross pathology of abdominal organs and blinded histological analysis of intestine, omentum, abdominal wall, and liver at three days after primary and booster immunization (arrows), respectively.

To assess the suitability of multiparameter telemetry for testing vaccine reactogenicity, we chose two experimental protocols of bovine herpesvirus 1 (BHV-1) vaccination (Fig. 1). In protocol #1, inactivated BHV-1 in complete Freund's adjuvant (CFA) was injected intraperitoneally (i.p.) for primary immunization (BHV-1/CFA) followed by a booster immunization in incomplete Freund's adjuvant (IFA) 28 days later (BHV-1/IFA). Protocol #2 consisted of one single intraperitoneal administration of live BHV-1 without addition of adjuvants. Protocol #1 reflected therefore a prototype vaccination regimen, which does not meet modern criteria for safety and tolerability, whereas apathogenic viral particle formulations as exemplified in protocol #2 are self-adjuvanted. Hence, they do not normally require the addition of chemical adjuvants and their tolerability compares favourably to the former one. The BHV-1-specific antibody response and IgG subclass repartition was monitored 2 weeks after primary immunization (day post immunization (dpi) 14) and booster vaccination (dpi 42), respectively, and demonstrated satisfactory immunogenicity of both protocols.

Antigen and vaccine formulations as well as methods and results for assessment of serum antibody response to BHV-1 can be found as supporting information (Text S1; Fig. S1).

To estimate potential adverse effects related solely to the injection procedures, animals were given sham injections two weeks before immunization (dpi –14). For this, the vehicle solution, containing neither BHV-1 nor adjuvant, was administered intraperitoneally. Telemetric transmitters were implanted prior to the sham injections to record in real-time and on the long-term not only heart rate but also interbeat interval, heart rate variability, body core temperature and locomotor activity. Recordings started one day before each injection to obtain baseline values and continued for three days after injection. On each of these days, a clinical assessment was performed, too. Additional groups of mice were subject to protocol #1 and #2, but were euthanized for macroscopic inspection and histological analysis of the abdominal organs three days after primary (dpi 3) and booster immunization (dpi 31), respectively.

### Clinical scoring fails to detect vaccine reactogenicity

For clinical scoring, sixteen mice (eight animals/protocol) were examined for appearance, posture, spontaneous and provoked movement and behaviour. In addition, body weight as well as food and water consumption were measured. These widely used parameters were chosen as read-outs of pain and discomfort or global impairment of health in small rodents. By these criteria, even a very thorough examination by an experienced veterinarian, specialized on small rodents, failed to detect abnormalities. None of the animals exhibited diarrhoea, ruffled fur, sunken flanks, enlarged abdomen, hunched posture, gait abnormalities, slowed movement, apathy or any other visible sign of discomfort.

**Figure 2:**
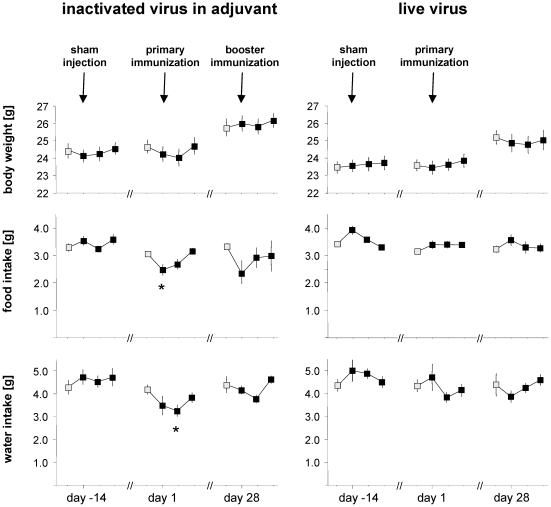
Changes in body weight, and food and water intake. Data were plotted over four days at sham injection, primary and booster immunization. The first data point of each series represents baseline values taken the day before an intraperitoneal injection (grey-filled symbols). Symbols indicate daily mean of eight mice per protocol, bars indicate SEM. Asterisks indicate statistical significance with p≤0.01.

A trend to decreased food and water consumption was noted in animals undergoing vaccination protocol #1 (Fig. 2). Food consumption was significantly reduced by 19+/−5% (mean+/−SEM; p = 0.008) on the first day after primary immunization. Water consumption decreased by 22+/−7% (mean+/−SEM; p = 0.009) on day 2 after primary immunization. Body weight showed a slight tendency to decrease during primary immunization in protocol #1. Conversely, not even a tendency of adverse effects was detected by these clinical parameters when assessing animals in protocol #2. It therefore seems likely that the above parameters could have detected reactogenicity of vaccination protocol #1, but only if analyzed in substantially larger groups of animals. This classical approach was therefore not well suited for high-throughput screening of vaccine reactogenicity.

### Organ pathology

**Figure 3:**
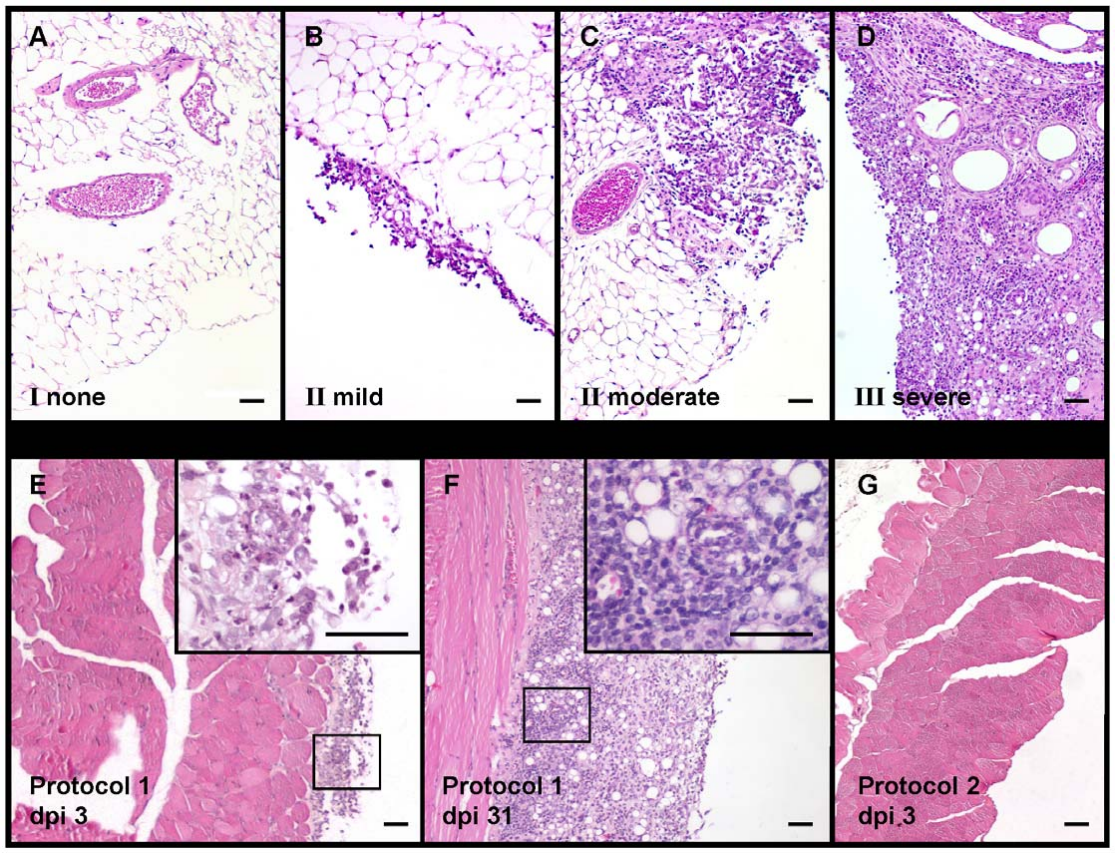
Histological grading of inflammation. (A–D) Representative examples of inflammation grades I–III in hematoxylin/eosin-stained sections of the omentum: Grade I: normal (A); Grade II: mild to moderate inflammation, with focal infiltrates (B, C); Grade: III severe inflammation, diffuse infiltrates (D). (E–G) Representative hematoxylin/eosin-stained sections of the abdominal wall from protocol #1 and protocol #2 at dpi 3 (3 days after primary immunization) (E, G) and from protocol #1 at dpi 31 (3 days after booster immunization) (F). Mild inflammatory infiltrates (Grade II) consisting mainly of neutrophils (inset) are visible in the serosa after primary immunization with inactivated virus in CFA (protocol #1, dpi 3) (E). After booster immunization with inactivated virus in IFA (protocol #1, dpi 31) the serosa of the abdominal wall is infiltrated with severe, diffuse inflammatory aggregates (Grade III) consisting mainly of lymphocytes and histiocytes (F). No abnormalities (Grade I) are detectable at 3 days after immunization with live virus (protocol #2, dpi 3) (G). Bars represent 50 µm.

Twenty-two mice, which had not received telemetry transmitters, were sacrificed on day three after primary (dpi 3) and booster immunization (dpi 31), respectively, to assess adverse effects at the injection site, i.e. in the peritoneal cavity. Gross macroscopic inspection of the abdominal organs failed to reveal any alterations. Conversely, microscopic analysis detected considerable pathological changes in liver, intestine, omentum and abdominal wall of the mice in vaccination protocol #1. Multifocal or even coalescing inflammatory infiltrates were mostly found in the fatty tissue of the omentum (Fig. 3, B–D) but also on the serosal surface of the liver and abdominal wall (Fig. 3, E, F). On day 3 after primary immunization with BHV-1/CFA, infiltrates consisted mainly of neutrophilic granulocytes (Fig. 3, E) consistent with acute, largely non-specific inflammation. Upon booster immunization with BHV-1/IFA, the infiltrates consisted mostly of lymphocytes and histiocytes, occasionally interspersed with multi-nucleated giant cells (Fig. 3, F) compatible with chronic and/or specific inflammation. In contrast, protocol #2 did not provoke any of these abnormalities (Fig. 3, A, G).

The occurrence and severity of histological changes were scored, revealing overall very significant differences between the two immunization protocols (Table 1, p = 0.0005). On day three after primary immunization with BHV-1/CFA (protocol #1), tissue alterations were mild to moderate. More severe changes were noted after boost with BHV-1/IFA (p = 0.002). In contrast, animals undergoing immunization with live BHV-1 (protocol #2) exhibited histopathological alterations of grade I, which was significantly lower than in protocol #1 (p = 0.003).

**Table 1:** Summary of local intra-abdominal side-effects.

histological grading		I (none)	II (mild to moderate)	III (severe)
**inactivated virus in adjuvant** (protocol 1)	3 days after primary immunization (dpi 3)		9/9	
**inactivated virus in adjuvant** (protocol 1)	3 days after booster immunization (dpi 31)		1/6	5/6
**live virus** (protocol 2)	3 days after primary immunization (dpi 3)	7/7		

In summary, histopathology detected vaccination-related inflammatory alterations, which were not readily born out when clinically assessing the animals. Adjuvants are, however, aimed at mimicking pathogen-associated molecular patterns (PAMPs), with the goal of eliciting inflammation and consequent immunostimulation at the site of injection. It can therefore be difficult to interpret the clinical significance of local histopathological changes, particularly with regard to systemic side-effects and tolerability.

### Telemetric measurements

We thus attempted to overcome the existing shortcomings in screening methods for safety and tolerability of vaccines. Mice were equipped with commercially available telemetry transmitters, allowing continuous long-term measurements of their heart rate, heart rate variability, body core temperature and locomotor activity while the animals are freely roaming. After implantation of the transmitter and a resting period, the mice underwent vaccination. Telemetric recording was performed over a period of 96 hours, and started 24 hours before administration of the vaccine to assess individual baseline values.

**Figure 4:**
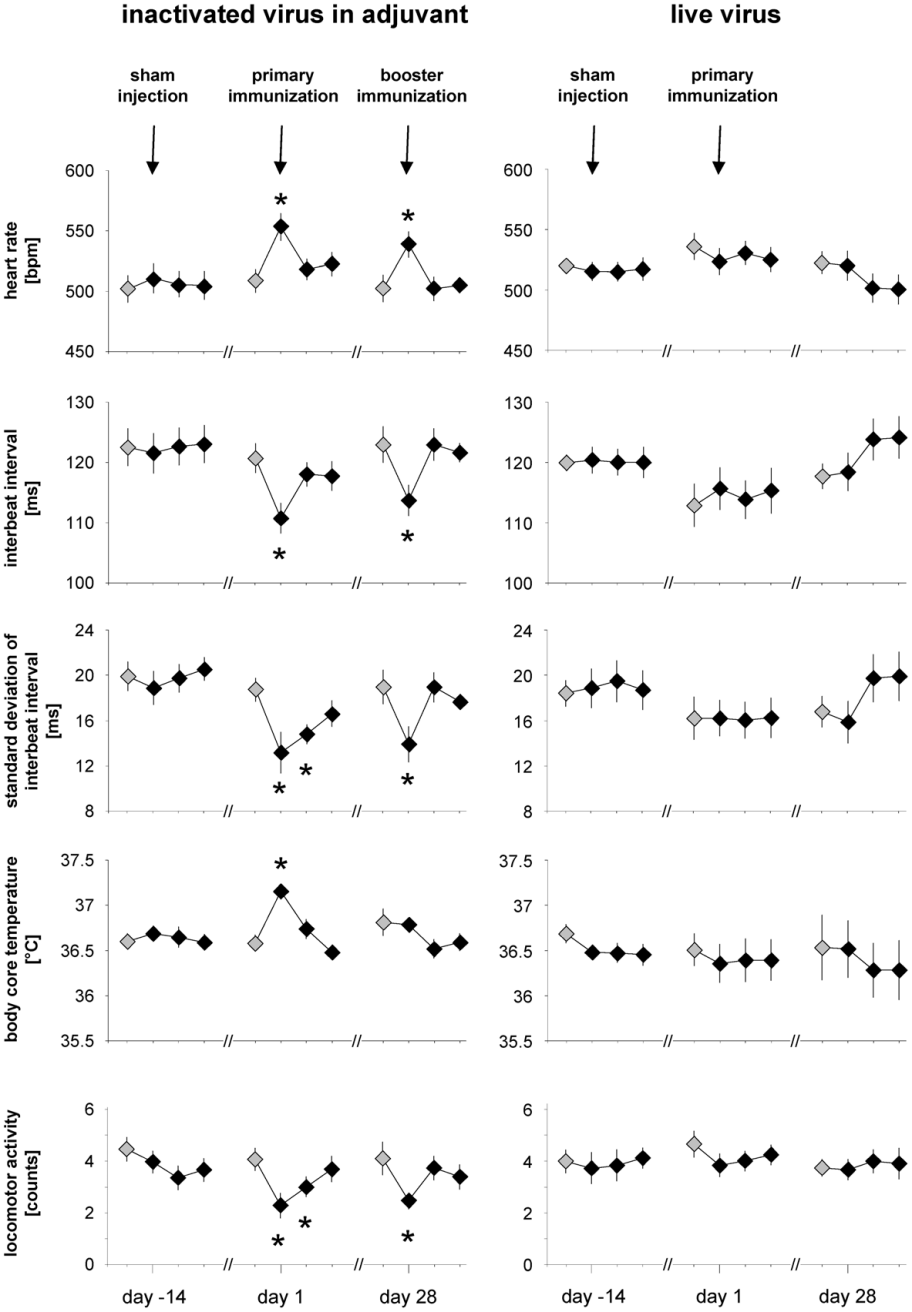
Telemetric assessment of vaccine reactogenicity. Heart rate [beats per minute, bpm], interbeat interval [milliseconds, ms], standard deviation of interbeat interval [milliseconds, ms], body core temperature [°C] and locomotor activity [counts] were recorded by telemetry and are displayed over three four-day periods at sham injection, primary and booster immunization, respectively (compare Fig. 1). The first data point of each series represents baseline values taken the day before an intraperitoneal injection (grey-filled symbols). Symbols indicate 24-hour means of eight mice per protocol, bars indicate SEM. Asterisks indicate statistical significance with p≤0.01. Note increased heart rate values with decreased interbeat interval and standard deviation of interbeat interval after primary and booster immunization with inactivated BHV-1 in Freund's adjuvant. Further, hyperthermia was noted after primary immunization, and depressed locomotor activity was recorded after primary and booster immunization with inactivated BHV-1 in Freund's adjuvant.

In animals undergoing vaccination protocol #1, heart rate increased, whereas the heart interbeat interval and heart rate variability (standard deviation of interbeat interval) decreased (Fig. 4). In mice, these alterations are typically seen as a result of pain or distress [19]. These changes on the first day after primary and secondary immunization were statistically highly significant with p between 0.01 and 0.0005. Perturbation of the standard deviation of interbeat interval persisted even throughout the second day after primary immunization (p = 0.001) and is commonly used as an indicator of sympathetic activation [20], and thus of a stress response [21]. Unlike vaccination protocol #1, protocol #2 consisting of live BHV-1 did not elicit significant alterations in any of the above parameters. Further, sham injection failed to affect heart rate, interbeat interval or standard deviation of the latter, confirming that neither the injection procedures nor the vaccine carrier solutions but the adjuvant accounted for the pathophysiological changes observed in protocol #1.

Hyperthermia, i.e. elevated body core temperature, is indicative of pyrogenicity, e.g. the effects of lipopolysaccharide and other pathogen-associated molecular patterns [22]. In vaccination protocol #1, hyperthermia was evident after the primary immunization in CFA, but not after the booster immunization in IFA (p = 0.003, Fig. 4). Whether hyperthermia was due to adjuvant or antigen or the combination thereof remains uncertain, but given the known reactogenicity profile of CFA, adjuvant likely has a contribution [23].

In accordance with the above parameters of the animal's wellbeing, telemetry detected also decreased locomotor activity in animals undergoing vaccination protocol #1. This alteration persisted for two days after primary immunization (p≤0.006) and for one day after booster vaccination (p = 0.007), whereas vaccination protocol #2 or sham injections failed to exert analogous effects (Fig. 4).

Interestingly, the telemetric parameters did not provide a direct quantitative correlate to the histopathological grading of inflammation i.e. the higher degree of histopathological inflammation upon secondary immunization (Table 1) did not augment disturbance of telemetric parameters (Fig. 4). This observation can serve as an example of the difficulty in correlating clinical manifestations with post-mortem observations on tissues, and provides further impetus for screening procedures such as telemetry, which involve the living animal.

## Discussion

The present study demonstrates the utility and sensitivity of telemetry for detection of adverse inflammatory effects of vaccine adjuvants. The comparison to more conventional approaches of clinical examination and histopathology has highlighted the obvious advantages of the method. By recording sympathetic activation, pyrogenicity and apathy in the free-ranging, untethered mouse, telemetry readily detected vaccine reactogenicity. Notably, these systemic side-effects of vaccination were not detected by clinical examination but were validated in histopathological analyses. The simultaneous detection of vaccine reactogenicity by multiple read-outs such as heart rate, interbeat interval, body core temperature and locomotor activity represents a clear advantage over more simple approaches with only one or two parameters recorded [24]. Not only are multiple parameters well suited to increase the sensitivity of the assessment, but they also can raise confidence in the result of an adverse reaction if multiple parameters evidence it. Albeit seemingly superior to classical methods of reactogenicity screening, future refinement of the methodology and parameters may further increase the sensitivity and specificity of the telemetry described herein. Accordingly it remains possible that e.g. live BHV-1 administration caused minor side-effects, which went undetected by the present methods. Whether or not such minor discomfort would translate into an acceptable clinical reactogenicity profile remains to be assessed in future work.

Laboratory mice are widely considered an optimal mammalian model organism for research and development of pharmaceutical products. The short reproductive cycle of mice, a high level of standardization owing to inbreeding and genetic homogeneity as well as ethical and economical considerations speak in its favour. In the context of compound reactogenicity and toxicity testing, however, these advantages were formerly often outweighed by the fairly limited information retrievable from clinical examination and observational assessment. Only severe symptoms of disease are readily reflected in appearance, posture and spontaneous behaviour. As prey animals, the mouse has supposedly evolved to hide distress in order to avoid attracting predators [25]–[27]. This behavior is also prompted by an investigator approaching the animal's cage. Moreover, merely the touching, handling or restraining – all of which are needed for detailed investigation – induce immediate alterations in heart rate, body core temperature [28], and blood pressure. These confounding factors have greatly complicated the reliable measurement of physiological parameters in mice, suggesting that data acquisition independently of an investigator's presence could offer an important step forward. Hence, telemetry as used in this study may provide the missing element to make the mouse model amenable to reactogenicity testing. Besides this key advantage, continuous reporting of data avoids any potential bias related to snapshot images in time. Additionally, continuous data recording allows various retrospective detailed analyses (e.g., circadian rhythmicity, differentiation between the night and daylight phase as shown in Fig. S2, and Fig. S3) and thus can reveal even subtle or transient alterations. Telemetry also is standardized, avoiding potential investigator-related variations in assessment. The possibility for automated real-time evaluation may be particularly relevant when it comes to screening of large compounds libraries, facilitating rapid decision-making by automated processes. Histopathology has confirmed the telemetric observations in this study and may in the future also provide an independent second line methodology to confirm screening results where needed. Similarly, larger animal models will remain a necessity for moving suitable candidate adjuvants towards clinical trials, not only for the assessment of their safety profile, but importantly also for immunogenicity and efficacy testing.

Taken together, this study demonstrates the utility of multiparameter telemetry in mice as a first-line screening approach for candidate vaccine adjuvant compounds. Using only limited numbers of mice, this method allows for their automated evaluation, differentiation and selection without sizeable risk for investigator-related bias. The early focussing on well tolerated test compounds and the possibility for up-scaling and partial automation in the industrial context bear considerable potential for rapidly advancing adjuvant discovery. This will not only help reducing animal testing, but, equally importantly, such methods can save precious time and will help channelling the limited financial resources available to more efficiently combat pressing global health problems.

## Methods

### Ethics statement

Ethical justification of the study as well as animal care and use were approved by the Cantonal Veterinary Office (Zurich, Switzerland) under license number 39/04. Housing and experimental procedures were in strict accordance with the Swiss animal protection law and in conformity with the European Convention for the protection of vertebrate animals used for experimental and other scientific purposes (Council of Europe nr.123 Strasbourg 1985). Housing and experimental procedures were also in accordance with the Guide for the Care and Use of Laboratory Animals (Institute of Laboratory Animal Resources, National Research Council, National Academy of Sciences, 1996).

### Mice: health status, housing and experimental conditions

Thirty-eight female mice of the strain 129Sv/Ev-IFNabRtmAgt (A129) [29] were randomly allocated to protocols and transmitter implantation or pathological analyses. These interferon receptor type I deficient mice were chosen owing to their widespread usage as highly virus-susceptible hosts, albeit fairly resistant to the BHV-1 infection used in this work [30]. Mice were free of all viral, bacterial, and parasitic pathogens listed in the recommendations of the Federation of European Laboratory Animal Science Associations [31]. Health status was confirmed by a health surveillance program throughout the experiment.

Mice were kept individually in filter top plastic cages (425 mm×266 mm×150 mm, floor area 820 cm^2^) with autoclaved dust-free sawdust bedding (80–90 g per cage) and autoclaved hay (8–10 g per cage) as nesting material. Each cage was equipped with a plastic shelter (Mouse house™, Indulab, Gams, Switzerland). Animals were fed a pelleted standard mouse diet (Kliba No. 3436, Provimi Kliba, Kaiseraugst, Switzerland) ad libitum, and had free access to sterilized drinking water. The light/dark cycle in the room consisted of 12/12 h (07:00–19:00) of artificial light. The climate was 21±1°C, with a relative humidity of 50±5%, and with 15 complete changes of filtered air per hour (HEPA H 14 filter).

All mice were bred and housed throughout the experiments in the same mouse room under biosafety conditions. The animal room was isolated from electronic noise and no disturbances (e.g. visitors or unrelated experimental procedures) were allowed. To avoid interfering influences, all necessary husbandry procedures were completed in the room three days before starting each measurement. All experimental procedures (i.e. injections, clinical scoring including weighing, blood sampling, necropsy) were carried out at the same time of day, 16.00 to 18:00 h, i.e. 1–3 hours before the onset of night phase in the animal room.

### Implantation of telemetric transmitters

Telemetric transmitters were implanted at 10 weeks of age. The TA10ETA-F20 transmitters (Data Sciences International, St. Paul, MN, USA), which are able to process heart rate, electrocardiogram, body core temperature, and locomotor activity in freely moving mice, were implanted as previously described [32]. Briefly, under anaesthesia with ketamine (Ketasol-100™, Graub, Bern, Switzerland), 45 mg/kg body weight, subcutaneously, and isoflurane (Isoflo™, Abbott, Baar, Switzerland) 3–5% in oxygen via nose mask, the telemetric transmitter body was implanted under aseptic conditions into the abdominal cavity. One telemetry lead was sutured as a wired loop to the xiphoid process. The other lead was tunneled subcutaneously from the thorax to the neck, where the wired loop electrode was fixed between the muscles located to the right of the trachea. Post-operative pain was treated with buprenorphine (Temgesic™, Reckitt and Colman Products Ltd., Hull, England), at a dose of 0.1 mg/kg bodyweight, injected subcutaneously twice per day for 4 days. After transmitter implantation, the mice had a period of 8 weeks convalescence until the start of the measurements and injection of the test compounds.

### Clinical scoring, acquisition of telemetric data and their evaluation

Clinical scorings and telemetric measurements were recorded in 4-day-periods for sham, primary and booster immunization respectively. The first day of each monitoring period was a control measurement, i.e. the day before the injection of a test compound represented baseline values. Recordings continued for 3 days following an intraperitoneal injection, to document the impact of the test substances.

Telemetric measurements were processed using the Dataquest LabPRO program, version 3.11 (Data Sciences International, St. Paul, MN, USA). The telemetric transmitter was switched on by touching the animal with a magnet; signals were detected by a receiver plate placed underneath the animal's cage.

Heart rate and electrocardiogram curves were recorded for 30 seconds every 5 minutes (sampling frequency 1000 Hz). Heart rate, interbeat interval and heart rate variability (standard deviation of interbeat interval) were established with commercially available software (Dataquest LabPRO program, version 3.11, Data Sciences International, St. Paul, MN, USA) by calculating the number of R-peaks and by determining the distance between R-peaks in the electrocardiogram curve. Electrocardiogram curves were subjected to a time domain analysis of heart rate variability as defined [20], [33], [34]: the interbeat interval and the standard deviation of interbeat interval were calculated from each segment measured. The standard deviation of interbeat interval was calculated as the standard deviation of interbeat intervals from normalized R-peaks in each 30-second measuring segment, and represents the variability in heart rate changes. Body core temperature was sampled for 10 seconds every 5 minutes. Locomotor activity was measured by the horizontal displacement of the animal in relation to two antennas in the receiver plate and was expressed in ‘activity counts’. Locomotor activity was recorded continuously and stored at 5 minute intervals. Telemetric data were calculated as mean of 24 hours.

Clinical scoring was carried out once daily. First, the animal's home cage was examined for any hints on health impairment (e.g., abnormal feces) or distress (e.g., unstructured cage area, poor nest building performance). If the animal was not hidden in that it was sleeping or resting in its nest, it was observed shortly in its home cage for aberrations of posture and spontaneous movement behaviour. Then, before weighing the animal, it was set on the cage lid to be investigated for clinical signs of illness or disease, such as diarrhoea, ruffled fur, abnormal posture, discharge of eyes, enlargement of abdomen, sunken flanks, or any other visible abnormalities. The subsequent weighing procedure normally provokes the mouse to express reactions such as flight or attention, which are particularly apparent after the animal is returned to its cage. Therefore, mice were observed for some seconds after weighing to register abnormalities in behaviour and movement, such as lameness, slowed movement or apathy.

Values of body weight, food and water consumption were established by weighing the animal, the food pellets and water bottle using a precision balance (PR 2003 Delta Range, Mettler-Toledo AG, Greifensee, Switzerland) especially designed to weigh moving animals. Body weights were corrected to take into account the weight of the transmitter (3.6 g). Daily values after intraperitonal injection of test compounds were compared with baseline values to identify the individual animals' changes in food and water consumption and change in body weight progression.

### Pathology

Nine mice of protocol #1 and 7 mice of protocol #2 were sacrificed by cervical dislocation and necropsied on day three after primary immunization (dpi 3). Six mice that had received primary and booster immunizations according to protocol #1 were necropsied 3 days after the booster immunization (dpi 31).

At necropsy the abdominal organs were macroscopically examined for any pathological changes. For histological investigation, the liver, samples of intestine, omentum and abdominal wall were fixed in 4% neutral buffered formalin and processed routinely. Tissue sections (2 µm) were stained with hematoxylin and eosin. Histopathological changes were evaluated in a scoring system ranging from I to III according to severity and distribution (I = no abnormalities, II = mild to moderate, III = severe; as exemplified in Fig. 3, A–D). Two pathologists independently evaluated each tissue samples in a blinded fashion.

### Statistical analysis

Results were analyzed statistically using the SPSS 17.0 software (SPSS Inc., Chicago, USA). Means and standard error of mean (SEM) were calculated for telemetric values, body weight, food and water consumption.

All data were tested for normal distribution with Q-Q plot and adapted Kolmogorov-Smirnov test. Homogeneity of variance was tested with Levene test. Measures of telemetric values, body weight, food and water consumption met the necessary assumptions for parametric analyses, while pathological results were not normally distributed and therefore tested with non-parametric tests. Thus, pathological results were analyzed with the Kruskal-Wallis H-Test for overall group comparison and Mann-Whitney U-Test for inter-group comparisons. Dependent t-tests for paired samples were used to analyze changes on days 1, 2, 3 after intraperitoneal injection in telemetric values, body weight, food and water consumption compared to the baseline values at one day prior to injection.

All tests were performed in two-sided manner. For multiple comparisons, Bonferroni correction was applied. Thus, significance for all statistical tests was established at alpha = 0.01 respectively p≤0.01.

## Supporting Information

Figure S1 **Antibody response to vaccination.** BHV-1 specific antibody titers of sera taken at dpi 14 (A: response to primary immunization) or dpi 42 (B: following booster vaccination in protocol #1, compare Fig. 1.) were determined by IgG subclass-specific ELISAs. Both live virus and inactivated virus in adjuvant induced an IgG2a-dominated antibody response indicative of a T helper 1-like immune response (A). Columns represent the mean+/−SEM of 6–7 mice per protocol. The dashed line indicates the limit of detection. Analysis of pre-immune sera confirmed that all mice were BHV-1-naive before immunization (data not shown). In each protocol, 1–2 individuals did not respond to primary immunization and were excluded from the analysis displayed in this figure.
(TIF)

Figure S2 **Time course of heart rate, interbeat interval, and standard deviation of interbeat interval at the night and light phase of each day.** Heart rate [beats per minute, bpm], interbeat interval [milliseconds, ms], and standard deviation of interbeat interval [milliseconds, ms] were recorded by telemetry and are displayed over three four-day periods at sham injection, primary and booster immunization, respectively (compare Fig. 1). The first data point of each series represents baseline values taken the day before an intraperitoneal injection. Symbols indicate 12-hour means of eight mice per protocol for the night and light phase of each day. Bars indicate SEM, asterisks indicate statistical significance with p≤0.01. Note increased heart rate values with decreased interbeat interval and standard deviation of interbeat interval after primary and booster immunization with inactivated BHV-1 in Freund's adjuvant.
(TIF)

Figure S3 **Time course of body core temperature and locomotor activity at the night and light phase of each day.** Body core temperature [°C] and locomotor activity [counts] were recorded by telemetry and are displayed over three four-day periods at sham injection, primary and booster immunization, respectively (compare Fig. 1). The first data point of each series represents baseline values taken the day before an intraperitoneal injection. Symbols indicate 12-hour means of eight mice per protocol for the night and light phase of each day. Bars indicate SEM, asterisks indicate statistical significance with p≤0.01. Note hyperthermia after primary immunization, and depressed locomotor activity after primary and booster immunization with inactivated BHV-1 in Freund's adjuvant.
(TIF)

Text S1 (DOC)
