# Supplementary figures and images for: Multiparameter Telemetry as a Sensitive Screening Method to Detect Vaccine Reactogenicity in Mice
Source: PLoS One. 2012 Jan 19;7(1):e29726. doi: 10.1371/journal.pone.0029726 (PMC3261877; doi:10.1371/journal.pone.0029726)

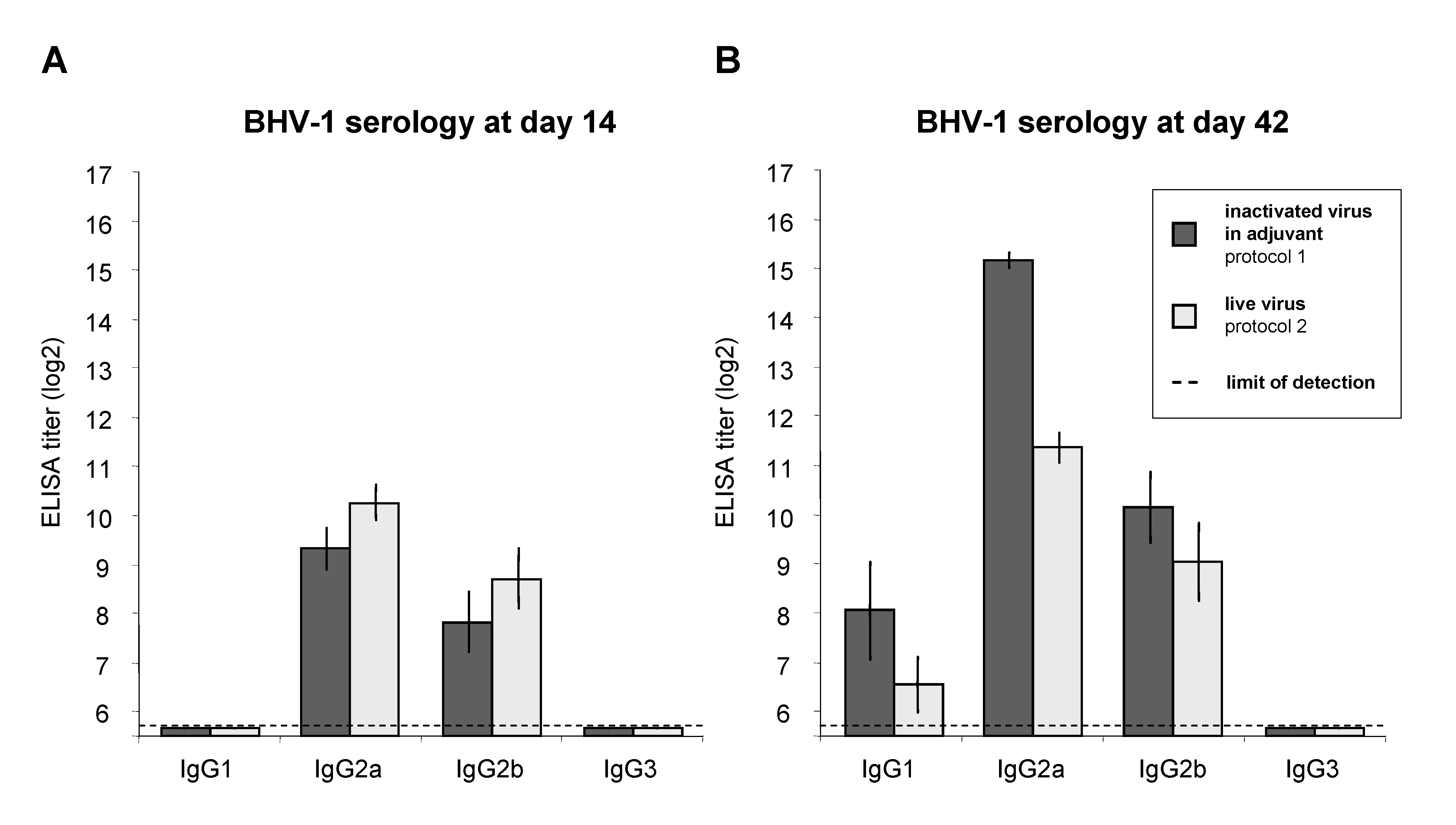

Supplement: Figure S1 — Antibody response to vaccination. BHV-1 specific antibody titers of sera taken at dpi 14 (A: response to primary immunization) or dpi 42 (B: following booster vaccination in protocol #1, compare Fig. 1.) were determined by IgG subclass-specific ELISAs. Both live virus and inactivated virus in adjuvant induced an IgG2a-dominated antibody response indicative of a T helper 1-like immune response (A). Columns represent the mean+/−SEM of 6–7 mice per protocol. The dashed line indicates the limit of detection. Analysis of pre-immune sera confirmed that all mice were BHV-1-naive before immunization (data not shown). In each protocol, 1–2 individuals did not respond to primary immunization and were excluded from the analysis displayed in this figure. (TIF) [file pone.0029726.s001.tif]

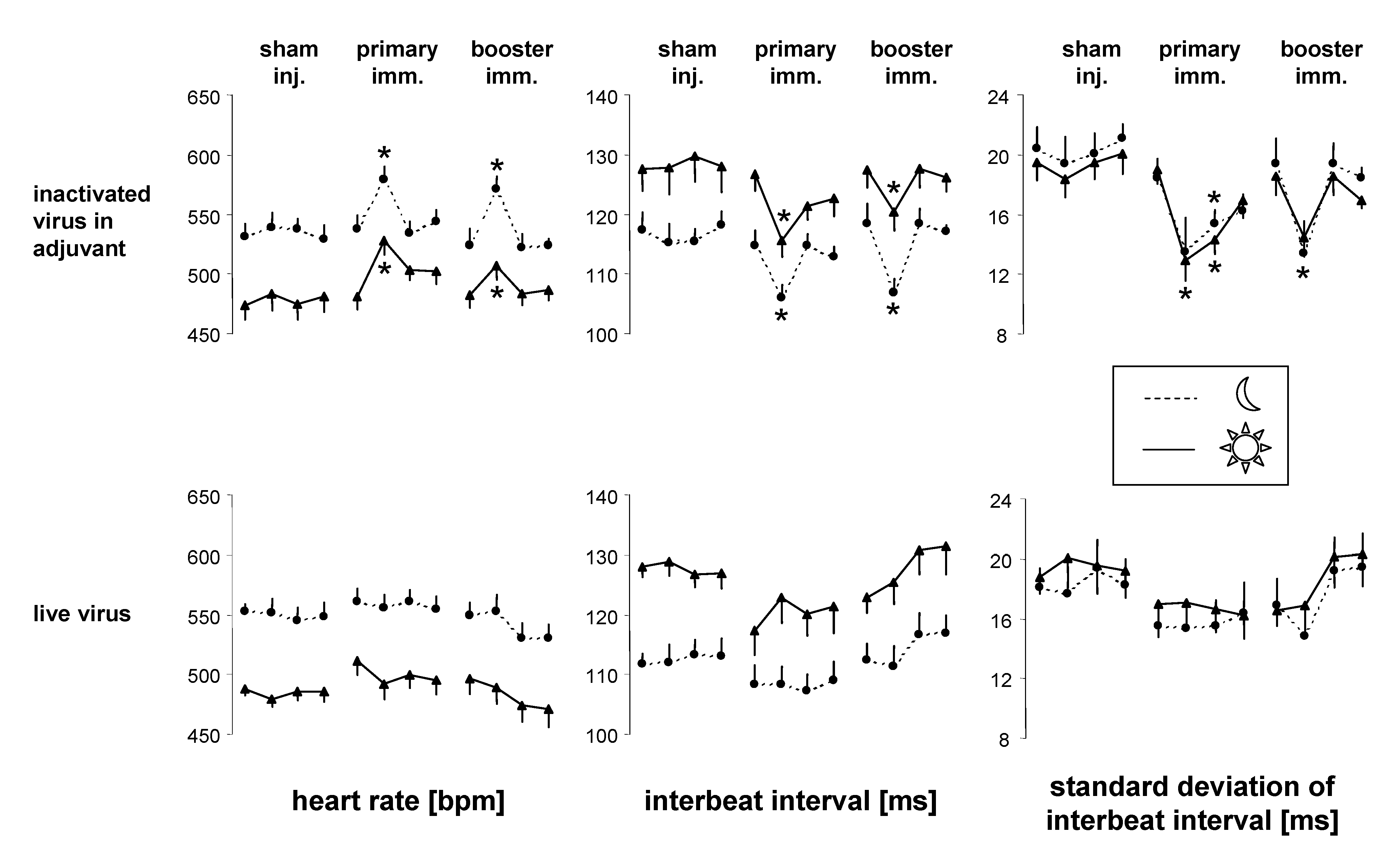

Supplement: Figure S2 — Time course of heart rate, interbeat interval, and standard deviation of interbeat interval at the night and light phase of each day. Heart rate [beats per minute, bpm], interbeat interval [milliseconds, ms], and standard deviation of interbeat interval [milliseconds, ms] were recorded by telemetry and are displayed over three four-day periods at sham injection, primary and booster immunization, respectively (compare Fig. 1). The first data point of each series represents baseline values taken the day before an intraperitoneal injection. Symbols indicate 12-hour means of eight mice per protocol for the night and light phase of each day. Bars indicate SEM, asterisks indicate statistical significance with p≤0.01. Note increased heart rate values with decreased interbeat interval and standard deviation of interbeat interval after primary and booster immunization with inactivated BHV-1 in Freund's adjuvant. (TIF) [file pone.0029726.s002.tif]

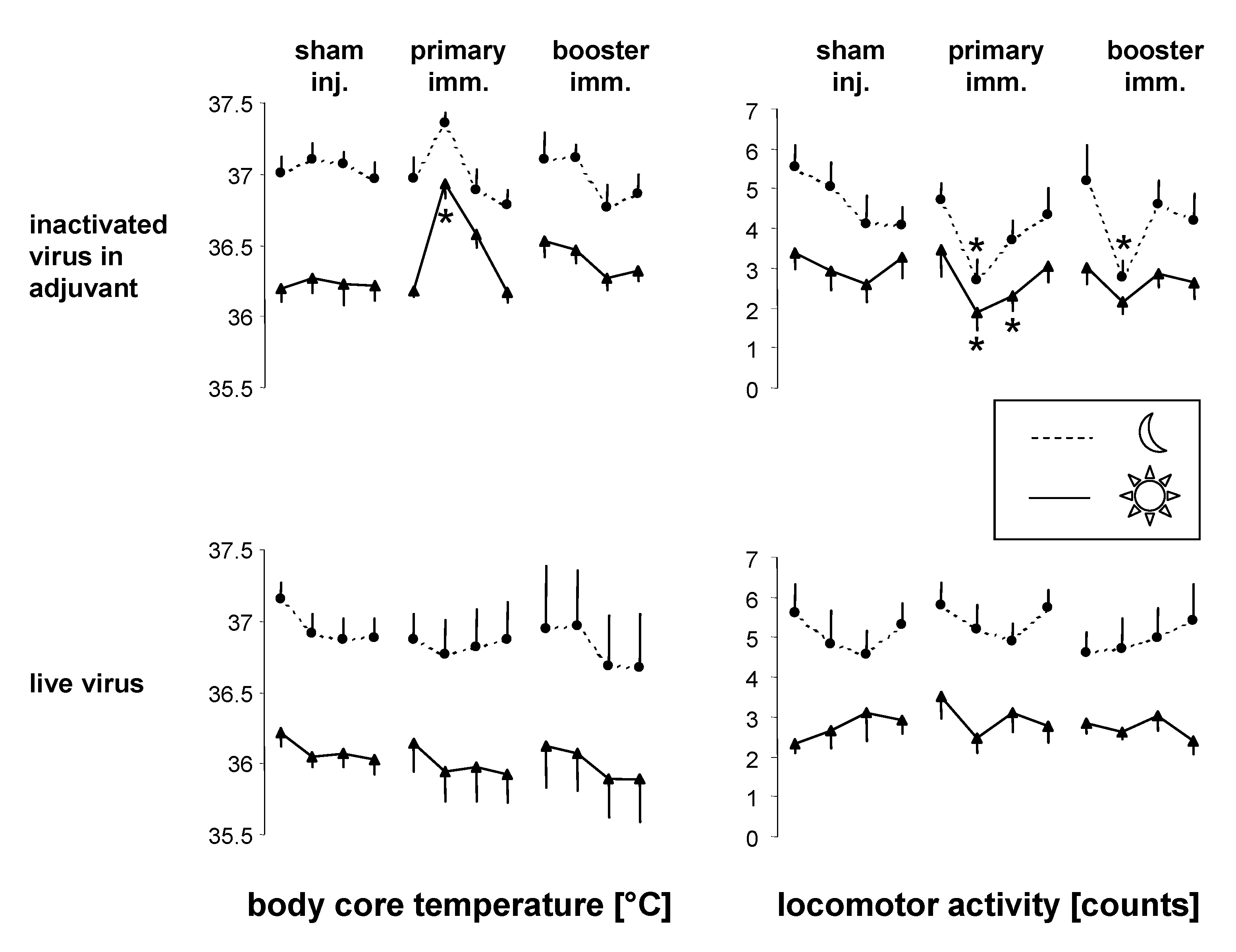

Supplement: Figure S3 — Time course of body core temperature and locomotor activity at the night and light phase of each day. Body core temperature [°C] and locomotor activity [counts] were recorded by telemetry and are displayed over three four-day periods at sham injection, primary and booster immunization, respectively (compare Fig. 1). The first data point of each series represents baseline values taken the day before an intraperitoneal injection. Symbols indicate 12-hour means of eight mice per protocol for the night and light phase of each day. Bars indicate SEM, asterisks indicate statistical significance with p≤0.01. Note hyperthermia after primary immunization, and depressed locomotor activity after primary and booster immunization with inactivated BHV-1 in Freund's adjuvant. (TIF) [file pone.0029726.s003.tif]
