# Supplementary material for: Multiparameter Telemetry as a Sensitive Screening Method to Detect Vaccine Reactogenicity in Mice
Source: PLoS One. 2012 Jan 19;7(1):e29726. doi: 10.1371/journal.pone.0029726 (PMC3261877; doi:10.1371/journal.pone.0029726)
Supplement: Text S1 — (DOC) [file pone.0029726.s004.doc]

**Supporting information**

## Antigen and vaccine formulations

As a model antigen we used bovine herpesvirus type 1 (BHV-1), either UV-inactivated or as live virus. BHV-1 strain Jura was grown and titrated on Madin Darby Bovine Kidney (MDBK) cells (ATCC, Rockville, MD, USA). The live virus used for immunization consisted of a cleared lysate of infected cells, which were maintained in Dulbecco’s modified Eagle medium (DMEM) (Gibco BRL, Life Technologies, Basel, Switzerland) supplemented with 2% fetal bovine serum (FBS; AMIMED, BioConcept, Allschwil, Switzerland), 100 units / ml Penicillin G (Sigma-Aldrich Corp. St. Louis, MO, USA) and 75 units / ml Streptomycin (Sigma-Aldrich Corp. St. Louis, MO, USA). For inactivation, the virus was treated with 600,000 µJ / cm^2^ 254-nm UV light in a UVC 500 Ultraviolet Crosslinker (Hoefer Pharmacia Biotech, San Francisco, CA, USA) and kept at 0°C during the inactivation procedure.

In protocol #1, immunization solutions consisted of UV-inactivated BHV-1 (corresponding to 10^7^ TCID_50_ live BHV-1) in 50 µl cell culture medium (DMEM supplemented with 2% FBS, 100 units/ml Penicillin G, and 75 units/ml Streptomycin). This was emulsified with 50 µl complete Freunds adjuvant (Sigma-Aldrich Corp. St. Louis, MO, USA, F-5881, Lot 014K8927) for primary immunization, or with 50 µl incomplete Freunds adjuvant (Sigma-Aldrich Corp. St. Louis, MO, USA, F-5506, Lot 112K8930) for booster immunization. 100 µl of the freshly prepared solution was injected intraperitoneally per individual.

In protocol #2, mice received an intraperitoneal injection of 10^7^ TCID_50_ of live BHV-1 in 100µl cell culture medium (DMEM supplemented with 1% FBS, 50 units/ml Penicillin G, and 37.5 units/ml Streptomycin).

For sham injections performed in the transmitter-bearing mice two weeks before immunization, the vehicle solution (100 µl, DMEM supplemented with 1% FBS, 50 units / ml Penicillin G, and 37.5 units / ml Streptomycin), containing neither BHV-1 nor Freunds adjuvant, was injected intraperitoneally.

## Assessment of serum antibody response to BHV-1

Approximately 100 µl of venous blood was taken under sevoflurane inhalation anaesthesia (Sevorane™, Abbott, Cham, Switzerland; 8% in 100% oxygen) from the retrobulbar venous plexus of the eye. The blood was allowed to coagulate and the serum harvested after centrifugation for 5 min at 1000 g and 4°C. The sera were then stored at –20°C. Pre-immune serum was taken at dpi –28, which is 2 weeks before the sham injection.

BHV-1-specific antibody titers were determined by IgG subclass-specific ELISA using 96-well microtiter plates coated with BHV-1 antigen and a negative control antigen (CHEKIT IBR/IPV B, Dr. Bommeli AG, Bern, Switzerland). Serial twofold dilutions of mice sera were made in PBS-T (0.1% Tween-20) starting at 1:100. Serum dilutions were added to the plate and incubated 1 h at 37°C. To detect antibodies bound to the ELISA plate, goat anti-mouse IgG1-, IgG2a-, IgG2b-, or IgG3-horseradish peroxidase (HRP) conjugated secondary antibodies (SouthernBiotech, Birmingham, AL, USA) were diluted 1:4,000 in PBS-T (0.1% Tween-20) and added to the ELISA plate. Again, plates were incubated 1 h at 37°C. The volume used for each step was 100µl per well and plates were washed three times with PBS-T between all steps. As substrate for the color reaction, ABTS [2,2'-azino-bis(3-ethylbenzthiazoline-6-sulphonic acid)] solution (containing 2mM ABTS, 78mM CH_3_COOH, 24mM NaCH_3_COO, 50mM NaH_2_PO_4_) was mixed with 1% H_2_O_2_ and added at 100µl per well. After incubation for 30 min at room temperature, the color reaction was stopped by addition of 50µl 10% dishwashing detergent (Handy; Migros, Zurich, Switzerland). The optical density at 405 nm (OD_405_) was read in a Multiskan RC microplate reader (Thermo Scientific, Waltham, MA, USA). The ∆OD_405_ between wells coated with BHV-1 antigen and wells coated with negative control antigen was calculated and used to determine BHV-1 specific antibody titers. Positive titers are indicated as reciprocal log_2_ value of the serum dilution at which the ∆OD_405_ was 0.1. Sera negative at the 1:100 dilution were scored as positive at a 1:50 dilution and this value (5.644) was defined as the limit of detection (baseline level).
